# Comorbidities, level of education, and alcohol consumption are predictive factors of undernutrition among adult outpatients living with diabetes: a case at Hawassa governmental hospitals in Ethiopia

**DOI:** 10.1017/jns.2025.21

**Published:** 2025-04-30

**Authors:** Asfaw Asegidew Meseret, Zelalem Tafese Wondimagegne

**Affiliations:** School of Nutrition, Food Science and Technology, Hawassa University, Hawassa, Ethiopia

**Keywords:** Complication, Concordant comorbidity, Diabetes mellitus, Diabetic complications, Ethiopia, Nutritional status, Predictors, T2DM, Type 2 diabetics mellitus, BMI, Body mass index, CKD, Chronic kidney disease, DM, Diabetics mellitus, HTN, Hypertension, CVD, Chronic vascular disease

## Abstract

Comorbidities, which are additional health conditions that occur alongside diabetes, can have a significant effect on blood sugar control. These conditions often complicate the management of diabetes and worsen overall health. Malnutrition, on the other hand, is a common concern for people with diabetes due to difficulties with food intake and metabolism. Proper nutrition is crucial for maintaining general health and effectively managing the disease. However, the extent of comorbidities and malnutrition within this group is not well understood in the study area. A cross-sectional study was conducted at Hawassa governmental hospitals between April and May 2023, involving 422 adult outpatients living with diabetes. The study aimed to evaluate their comorbidities, nutritional status, and associated factors. The required data were collected using structured and semi-structured questionnaires. Bivariate and multivariate logistic regression analyses were conducted using SPSS version 25.0. Undernutrition and concordant comorbidities were prevalent in the study population, occurring at rates of 15.2% and 57.8%, respectively. Additionally, 18.5% of participants were classified as overweight and obese with a BMI greater than 25 kg/m^2^. Three significant predictors of undernutrition among adult outpatients living with diabetes were identified: alcohol intake (P < 0.05), comorbidities (P < 0.01), and educational status (P < 0.05). Concordant comorbidity was notably common in these patients. It is recommended that the healthcare system consider comorbid conditions when managing diabetes. A longitudinal study is suggested to provide stronger evidence on these findings.

## Background

Diabetes is a common, chronic metabolic disease that is characterised by elevated blood glucose levels. Type 1 diabetes is caused by insufficient insulin, whereas type 2 diabetes is caused by insufficient insulin and insulin resistance.^(1)^ More than 90% of diabetes cases worldwide are type-2 diabetes, which primarily affects adults and is brought on by the body becoming resistant to insulin or by the pancreas producing insufficient amounts of the hormone.^(2)^ Diabetes is one of the diseases with the fastest rate of global increase.^(3)^ It is a difficult public health issue with major health repercussions and expenses linked to an unhealthy lifestyle, including cardiovascular illnesses, amputations, vision loss, and renal failure.^(4)^

The World Health Organization (WHO) stated that during the past ten years, low- and middle-income countries have seen a faster increase in the prevalence of diabetes.^(5)^ Approximately 75% of people with diabetes worldwide reside in low- and middle-income nations.^(6)^ Despite significant regional and national variations in the trend and severity of NCD risk factors, 28 million people are expected to have the disease by 2030, with T2DM (Type 2 diabetics mellitus) being most common in Africa, where the current number of people living with diabetes has reached 19 million.^(7)^ This figure aligns with ongoing trends of increasing diabetes prevalence in the region, which has been attributed to factors like urbanisation, lifestyle changes, and limited access to early diagnosis and treatment.

Similarly, due to its sizable population, Ethiopia has the highest rate of diabetes prevalence in Africa, ranging from 2.0% to 6.5%, with a low of 2% in smaller rural areas.^(8)^

Diabetes can have a negative impact on a person’s quality of life. If blood glucose levels are not controlled, hyperglycaemia is a common side effect of uncontrolled diabetes that can cause major harm to numerous bodily systems over time, particularly to the blood vessels and nerves.^(9)^ Comorbidities and undernutrition are common challenges faced by individuals with diabetes. Comorbidities, such as cardiovascular diseases, hypertension, and kidney dysfunction, often occur alongside diabetes and can complicate disease management. Concordant and discordant comorbidities are two categories of chronic conditions that are commonly present in patients with diabetes.^(11)^ A recent review synthesising evidence on the burden of diabetes noted the increasing prevalence of comorbidities, highlighting that many individuals with diabetes are also affected by complications like cardiovascular disease and kidney dysfunction.^(10)^ Concordant comorbidities are defined as two or more diseases that share similar pathophysiological traits and are more likely to be influenced by the same underlying factors, often requiring a unified approach to management. In contrast, discordant comorbidities refer to diseases with distinct and unrelated pathophysiological features.^(12)^ Thus, among patients with diabetes, concordant comorbidities such as obesity, hypertension, hyperlipidaemia, chronic vascular disease (CVD), and chronic kidney disease (CKD) are frequently reported.^(13)^ An Indian study found that 84% of diabetic patients had one or more comorbid conditions, with hypertension being the most common chronic comorbidity in this primary care population.^(14)^ A retrospective cohort study conducted to quantify the prevalence and co-prevalence of common comorbidities among type 2 diabetes found that the majority of adults with diabetes have at least one comorbidity.^(15)^ A study conducted in Nigeria, Ghana, and Kenya found that comorbidity rates among type 2 people living with diabetes in tertiary health centres are rapidly increasing, ranging from 6% to 64%, likely due to differences in healthcare access, socioeconomic factors, and lifestyle or genetic influences.^(16)^ A study conducted in eastern Ethiopia reported that 55.8% of people living with diabetes had concordant comorbid conditions.^(17)^ Undernutrition is also common among people living with diabetes, with a recent study reporting a pooled prevalence of 20.5% among people living with diabetes in Ethiopia.^(18)^ Being underweight is a recognised risk factor for diabetic complications, as confirmed by a Korean study showing that underweight people living with diabetes had more than twice the risk of cardiac complications during follow-up.^(19)^

People with diabetes are frequently advised to adopt healthy eating practices worldwide because diet control is thought to be the most important aspect of diabetes treatment.^(20)^ Undernutrition, marked by insufficient intake of essential nutrients, is common among diabetic patients and can worsen their health and glycemic control, as they often struggle to find a diet that is both nutritionally adequate and sufficient in quantity.^(21)^ Patients’ understanding of a recommended diet has an impact on their choices of foods and eating habits. For this reason, dietary evaluation is crucial for promoting health, preventing disease, and developing personalised treatment plans for diabetic patients.^(22)^ It has been demonstrated that dietary interventions for people living with diabetes improve functional outcomes while reducing mortality and complications.^(23)^ Both comorbidities and undernutrition can degrade the quality of life and heighten the risk of complications in diabetic patients, emphasising the need for early detection and comprehensive management. Previous research has shown a significant rise in diabetes-related morbidity and mortality in sub-Saharan Africa.^(24)^ Ethiopia is among the African countries with a significant diabetes burden, affecting 1.7 million adults.^(25)^ Most people living with diabetes have reported inadequate control over their blood sugar levels, indicating a need for improved management strategies and support.^(26)^ To develop appropriate plans and health programmes, there is a need to shift healthcare priorities and update current data on the prevalence and complications of diabetes in Ethiopia.^(27)^ However, there is a notable lack of data regarding the risk factors and epidemiology of diabetes comorbidities.^(17)^ The current study was designed to assess the comorbidities, nutritional status, and associated factors among individuals with diabetes. The findings aim to contribute valuable insights for developing targeted recommendations, including nutritional care interventions. By providing evidence-based data, the study holds the potential to enhance diabetes care and control complications, ultimately improving patient outcomes and public health strategies.

## Methods

### Study design and setting

This study has been conducted and reported in accordance with the STROBE guidelines for observational studies (Elm *et al.*, 2014).^(28)^ A cross-sectional study was conducted from May 2022 to April 2023 across several healthcare institutions, including Adare General Hospital, Hawassa University Comprehensive Specialized Hospital, and the governmental hospitals in Hawassa City. Located 273 km north of Addis Ababa, Hawassa serves as a key site for diabetes care, with hospitals providing comprehensive treatment and regular follow-up visits at least once a month. Approximately 2,600 individuals with diabetes were being monitored at these hospitals. All patients aged 18–65 years enrolled in the outpatient diabetes management programme who agreed to participate were included in the study.

### Measurements

The data collection tools used in this study included a digital scale (770 alpha), a stadiometer, a strain-resistance metre, and a structured questionnaire. The questionnaire gathered information on sociodemographic factors, behaviours, health-related issues, household food security, dietary diversity, and anthropometric measurements (weight and height). Information on comorbidities for individuals with diabetes was obtained from patient charts. Glycemic control was evaluated by averaging the last three fasting blood glucose readings, with a normal fasting blood sugar level defined as below 100 milligrams per decilitre. Sociodemographic data were collected via structured questionnaires, face-to-face interviews, and physical measurements using standardised methods and calibrated equipment.

Height was measured with participants standing barefoot and without shoes, ensuring their body was upright with their buttocks, scapula, and head in contact with the stadiometer. The reading was recorded to the nearest 0.1 cm, and the measurement was taken twice, with the average used for analysis. Participants were then weighed in light clothing and barefoot, with the weight recorded to the nearest 0.1 kg. To minimise measurement errors, the instruments were calibrated after each use. A scoring system was developed based on participant responses to assess comorbid conditions, household dietary diversity, and food insecurity. The food insecurity section included nine statements with ‘yes’ or ‘no’ responses, which were scored as follows: ‘1’ for infrequent (once or twice), ‘2’ for occasional (three to ten times), and ‘3’ for frequent (more than ten times). ‘No’ responses received a score of ‘0’. This scoring system categorised household food insecurity into four levels: food secure, mild, moderate, and severely food insecure, as outlined by the Food and Nutrition Technical Assistance guidelines.^(29)^

The Food and Agriculture Organization of the United Nations employed a standardised tool to assess dietary diversity, which involved counting the number of food groups consumed the previous day from a set list of 12. The dietary diversity score was then calculated and categorised as low (<4), moderate (4–5), or high (>5).^(30)^ Cigarette smoking was assessed based on participants’ self-reported smoking history within the two weeks prior to the survey. Study participants were classified as Type 1 or Type 2 diabetes based on data directly extracted from the patient registry.

We utilised the short version of the International Physical Activity Questionnaire (IPAQ), a widely recognised tool designed to evaluate individuals’ physical activity levels based on their activities over the past week. Through this questionnaire, we inquired about the participants’ frequency and duration of walking, moderate-intensity activities, and vigorous-intensity activities. The IPAQ classifies physical activity levels according to the intensity, frequency, and duration of activity, categorising individuals as ‘inactive’, ‘insufficiently active’, or ‘sufficiently active’. For this study, we simplified the classification by grouping ‘inactive’ and ‘insufficiently active’ as ‘not active’, while those classified as ‘sufficiently active’ were considered ‘active’.^(31)^

### Sample size and sampling technique

Sample sizes for three specific objectives were calculated: prevalence of comorbidities, nutritional status and factors associated with nutritional status among adult outpatients living with diabetes using a uniform population proportion formula with a 95% confidence interval and a 5% margin of error (d). We used the assumption of comorbidity from a study in eastern Ethiopia,^(17)^ the nutritional status of Vietnamese people living with diabetes,^(32)^ and a maximum ratio of 0.5 for factors associated with the nutritional status of adult people with diabetes. The final sample size of 422 was calculated by adding a 10% non-response rate. Participants were selected through systematic random sampling from a group of 2,593 individuals with diabetes receiving treatment at two public hospitals. The sample size was proportionally divided between 109 patients from Adare General Hospital and 313 from Hawassa University General Specialist Hospital, based on the total number of adults with diabetes at each hospital. The first participant was randomly chosen using a lottery method, and then every sixth individual was selected from the sampling frame, following the sampling interval (k = 6).

### Study variables

#### Dependent variable

The nutritional status of adult outpatients living with diabetes as measured by BMI, served as the dependent variable. It was computed using heights in metres and body weight in kilograms (kg/m^2^). Patients were considered undernourished if their BMI was less than 18.5 kg/m^2^. The WHO classifies BMI as follows: a BMI of <16.0 kg/m² is considered severe thinness, 16.0–16.99 kg/m² is moderate thinness, and 17.0–18.49 kg/m² is mild thinness.^(33)^

#### Independent variables

A questionnaire was administered through face-to-face interviews to collect data on sociodemographic factors such as age, sex, marital status, income, religion, and education level. The participants’ responses were then analysed to assess their associations with comorbid conditions.

The dietary diversity score (DDS) was developed by asking study participants to recall their food intake over a 24-hour period. High dietary diversity (≥ 6 food groups) included cereals, green leafy vegetables, vitamin A-rich fruits, oil, other vegetables, fish, legumes, nuts, and seeds. A medium level of dietary diversity was defined as consuming four to five food groups, such as cereals, leafy green vegetables, vitamin-rich fruits, and oil. A diet with three or fewer food groups was considered the least diverse, including only green leafy vegetables, vitamin A-rich fruits, and oil.^(30)^ The Household Food Insecurity Access Scale (HFIAS) questionnaire was employed to assess the level of food insecurity within households of study participants. The nine-item HFIAS questionnaire was scored according to the Indicator Guide.^(29)^ Health-related information, including the type of diabetes, duration since diagnosis, and glycemic control, was retrieved from the individual patient charts.

### Data quality control

Data collectors underwent two-day training on the tool components, including the Kobo Toolbox, data collection methods, and procedures, prior to the actual data collection. The collected data were rigorously reviewed for completeness, accuracy, and consistency each day during the data collection period. At the end of each day, supervisors reviewed the questionnaires to ensure their completeness.

### Data analysis and procedures

The collected data was coded, recoded, cleaned, and examined to identify outliers and missing values, ensuring completeness through manual checks. It was then exported from the Kobo toolbox to SPSS version 25.0. After data exploration, descriptive statistics were used to analyse variables such as the sociodemographic characteristics of the participants in relation to the dependent variable. A chi-square test was conducted to identify independent variables associated with the dependent variable. To evaluate the goodness of fit of the final model, the Hosmer and Lemeshow test and log-likelihood were applied. A binary logistic regression model was used to determine factors linked to nutritional status. Variables with a P-value ≤ 0.25 in the bi-variable logistic regression were included in the multivariable logistic regression analysis. An adjusted odds ratio (AOR) with a 95% confidence interval (CI) was used to measure the strength of the association, with a P-value ≤ 0.05 indicating statistical significance in the multivariable logistic regression. Multicollinearity was assessed, and the highest variable inflation factor recorded was 1.64, indicating no threat of multicollinearity.

## Results

### Socio-demographic characteristics of study participants

A 100% response rate was achieved from the 422 adult outpatients living with diabetes who participated in the study, with medical record reviews and interviews conducted. The participants had an average age of 44.46 ± 14.33 years, and 204 (48.3%) were between the ages of 35 and 54. Among the participants, 175 (41.4%) had a college degree or higher, while 115 (27.3%) had no formal education. As shown in Table 1, the majority of participants were married (353, 81.8%), and 109 (25.6%) were employed in government positions.

**Table 1.** Socio-demographic behavioural, and clinical characteristics of adult diabetic outpatient at Hawassa governmental hospitals, 2023 (n = 422)

Variables	N (%)
Age in years	
18–24	47 (11.1)
28–34	56 (13.3)
35–54	204 (48.3)
≥55	115 (27.3)
Sex	
Male	230 (54.5)
Female	192 (45.5)
Region	
Sidama	123 (29.1)
Amhara	115 (27.3)
Oromo	100 (23.7)
Gurage	54 (12.8)
Other**	30 (7.1)
Marital status	
Single	41 (9.7)
Married	353 (83.6)
Divorced	14 (3.3)
Widowed	14 (3.3)
Occupation	
Government employer	109 (25.8)
House wife	104 (24.6)
Daily labourer	9 (2.1)
Self-employee	104 (24.6)
Other*	96 (22.6)
Average monthly income ETB	
<3000	96 (24.4)
3000–5999	58 (14.7)
≥6000	136 (34.6)
Educational status	
No formal education	115 (27.3)
Primary education	63 (14.9)
Secondary education	69 (16.4)
College and above	175 (41.4)
Physically active	
Yes	216 (51.2)
No	206 (48.8)
History of cigarette smoking in the two weeks before the survey	
Yes	77 (18.3)
No	345 (81.7)
History of alcohol intake in the two weeks before the survey	
Yes	134 (31.8)
No	288 (68.2)
History of meal skipping in the two weeks before the survey	
Yes	248 (58.8)
No	174 (41.2)
Types of diabetes	
Type I	99 (23.5)
Type II	323 (76.5)
Types of treatment	
Insulin	88 (20.9)
Oral hypoglycaemic agent	223 (52.8)
Both	108 (25.6)
Condition of glycemic control	
Controlled	240 (56.9)
Uncontrolled	182 (43.1)
Duration since diagnosed as diabetic	
≤ 5 years	172 (40.8)
>5 years	250 (59.2)

1 USD = 59 ETB, Other** = wolaita, Hadiya, Other* = house wife, student.

### Behavioural characteristics, and clinical conditions of the study population

Among the respondents, 77 (18.2%) reported being smokers, and 134 (31.8%) consumed alcohol. The average duration since their diabetes diagnosis was 6.54 years. The majority of participants, 323 (76.5%), were diagnosed with Type 2 diabetes, 223 (52.8%) were currently using oral hypoglycaemic agents, and 216 (51.2%) were physically active. Furthermore, 228 (54.0%) of the participants had poor glycemic control (Table 1).

### Prevalence of comorbidity

**Fig. 1.**
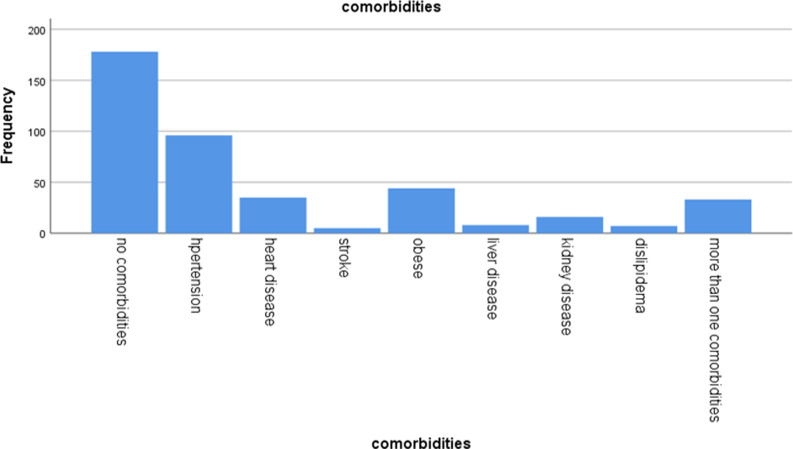
Comorbid disease distribution among adult outpatients living with diabetes at Hawassa government hospitals, 2023 (n = 422).

The overall prevalence of concordant comorbidities among adult outpatients living with diabetes was 245 (58%). Among these, hypertension was reported by 96 (22.7%) participants, obesity by 44 (10.4%), heart disease by 35 (8.3%), dyslipidemia by 7 (1.7%), chronic kidney disease by 16 (3.8%), and stroke by 5 (1.2%). Additionally, 32 (7.6%) patients had more than one comorbidity (Fig. 1).

### Dietary and nutritional status of respondents

According to the HFIAS, 171 (40.5%) of the adult outpatients living with diabetes were from food-insecure households. Analysis of the food groups consumed by the participants on the day prior to the survey revealed a mean (± SD) dietary diversity score of 6.2 (±1.4). Over three-fourths of the participants had consumed foods made with oils, fats, or butter (97.4%), cereals (90%), and white roots and tubers (80.5%). However, the intake of nutrient-dense foods, such as animal-source foods and fruits and vegetables, was relatively low. Overall, 200 (47.4%) participants had low dietary diversity. The prevalence of undernutrition among adult outpatients living with diabetes was 15.2% (BMI < 18.5 kg/m²) (Table 2). Specifically, 38 (9%) were mildly underweight (BMI 17.0–18.49 kg/m²), 8 (1.9%) were moderately underweight (BMI 16.0–16.99 kg/m²), and 18 (4.27%) were severely underweight (BMI < 16.0 kg/m²). Additionally, 78 (18.5%) individuals were overweight or obese (BMI > 25 kg/m²), while 280 (66.4%) were of normal weight (BMI 18.5–24.9 kg/m²).

**Table 2.** Dietary and Nutritional characteristics counselling among adult diabetic outpatient at Hawassa governmental hospitals, 2023 (n = 422)

Variables	N (%)
House hold food insecurity	
Secured	251 (59.5)
Mild food insecure	80 (19.0)
Moderate food insecure	51 (11.0)
Severely food insecure	40 (9.5.0)
Dietary diversity	
Low(<6)	200 (47.4)
High (≥6)	222 (52.6)
Types of food items consumed	
Food made with oils, fat or butter	411 (97.4)
Condiments	397 (94.1)
Cereals	380 (90.0)
White roots and tubers	340 (80.5)
Legumes or nuts	221 (52.4)
Any sugar/honey	203 (48.1)
Other fruits or vegetables	130 (30.8)
Vitamin A rich fruits and vegetables	126 (29.9)
Milk/milk products	121 (28.7)
Eggs	120 (28.4)
Fish	97 (23.1)
Any meat (excluding fish)	86 (20.4)
Nutritional counselling in the last one month	
Received	135 (32.0)
Not received	287 (68.0)
BMI of study participants	
Severe thinness	18 (4.3)
Medium thinness	8 (1.9)
Mild thinness	38 (9.0)
Normal	280 (66.4)
Overweight and obese	78 (18.5)

### Predictors of undernutrition

The multivariable analysis’s candidacy criteria were met by the history of alcohol consumption, duration of time after being diagnosed as diabetic, dietary diversity, educational status, presence of comorbidities, glycemic control, and cigarette smoking (P < 0.25), according to the binary logistic regression analysis. However, the final model found that the presence of comorbidities, educational attainment, and alcohol consumption history were significantly linked to undernutrition. Study participants who reported alcohol consumption were nearly twice as likely to be undernourished (AOR = 1.91, 95% CI: 1.08–3.38, P < 0.05) compared to those with no history of alcohol use. When comparing respondents with no formal education to those with higher education, the odds of developing undernutrition were more than three times higher (AOR = 3.12, 95% CI: (1.56–6.23), at P < 0.01). Adult outpatients living with diabetes who had at least one comorbidity were more than twice as likely to be undernourished as those who did not (AOR = 2.55, 95% CI: (1.38–4.71), at P < 0.01) (Table 3).

**Table 3.** Summary of logistic regression analysis for factors associated with under nutrition among adult diabetic outpatient at Hawassa governmental hospitals, 2023 (n = 422)

Variables	Undernutrition		COR 95% CI	AOR 95% CI	P value
	No	Yes			
History of alcohol intake					
Yes	37	27	1.71 (0.99, 2.95)	1.91 (1.08, 3.38)*	0.024
No^a^	251	107	1	1	
Duration of diabetes, yrs					
≤ 5 years	24	40	1.17 (0.67, 2.03)	1.27 (0.71, 2.27)	0.343
>5 years	148	210	1	1	
Dietary diversity					
≤5	196	26	1.76 (1.02, 3.03)*	1.58 (0.90, 2.77)	0.174
>6	162	38	1	1	
Educational status					
Secondary school	58	11	1.88 (0.82, 4.29)	1.90 (0.82, 4.39)	
Primary school	52	11	2.10 (0.91, 4.81)	2.31 (0.99, 5.42)	
No formal education	82	26	2.90 (1.47, 5.69)*	3.12 (1.56, 6.23)**	0.001
College and above	159	16	1	1	
At least one comorbidity					
Yes	16	48	2.45 (1.34, 4.48)**	2.55 (1.38, 4.71)**	0.003
No	161	197	1	1	
Controlled sugar level					
Yes	194	46	0.46 (0.25, 0.82)*	0.57 (0.30, 1.07)	
No	164	18	1	1	0.034
History of cigarette smoking					
Yes	296	49	1.46 (0.77, 2.77)	1.10 (0.51, 2.38)	0.794
No	26	15	1	1	

Abbreviations: AOR, adjusted odd ratio; COR, crude odd ratio; BMI, body mass index.

* Statistically significant P <.05; ** Statistically significant P <.001.

^a^ Reference catagory.

## Discussion

This study aims to assess the prevalence of undernutrition and comorbidities among adult outpatients living with diabetes, as well as the factors influencing them. Our findings confirmed a high prevalence of undernutrition and associated comorbidities among adult outpatients living with diabetes in the study area. Specifically, the results showed that 245 (58%) of outpatients living with diabetes had concordant comorbidities. While this result is significantly greater than the comorbidity found in previous research from Ethiopia and Bangladesh,^(34,35)^ it is consistent with the findings of an earlier Ethiopian investigation.^(17)^ Conversely, our result was substantially lower than those of previous studies conducted in other parts of the world, including Switzerland (91%),^(36)^ Spain (82%),^(37)^ and India (84%).^(38)^ The observed variation in comorbidity prevalence among studies may be attributed to changes in the sociodemographic characteristics of the study populations as well as variations in the types of diabetes that were included in the investigations.

The study found that 15.2% of adult outpatients living with diabetes were undernourished, a figure lower than the 43.1% prevalence reported in another study conducted in Ethiopia.^(39)^ This difference could be explained by variations in sociodemographic factors, lifestyle, economic status among the study populations, and the possibility of underdiagnoses. The current study found that adult diabetic patients with a history of alcohol consumption were almost twice as likely to experience undernutrition. This finding aligns with a previous study in Uganda, which reported a 23.45% prevalence of alcohol consumption among individuals with diabetes, highlighting the need for interventions to address the impact of alcohol use in this population.^(40)^ Although alcohol consumption negatively affects the nutritional status and treatment outcomes of diabetic patients, alcohol consumption by diabetics can worsen blood sugar control in those patients. Additionally, long-term alcohol ingestion by adult outpatients living with diabetes who are not adequately nourished can lead to dangerously low blood sugar levels.^(41)^ This can affect nutritional status and increase the risk of diabetes-related medical complications.

Educational attainment is a key factor influencing the nutritional status of adults, as individuals with higher levels of education are more likely to make informed dietary choices, engage in health-promoting behaviours, and access healthier food options.

Consistent with findings from similar studies, educational attainment is a key factor influencing the nutritional status of adults.^(42–44)^ Our findings indicate that individuals with diabetes who have no formal education are more than three times as likely to suffer from undernutrition compared to those with a college degree or higher. Educational attainment is a key determinant of adults’ nutritional status. People with higher education levels are generally more knowledgeable about the benefits of a balanced diet and are better equipped to make a healthy food choice, which contributes to improved nutrition. On the other hand, those with limited education often lack awareness about essential nutrients and may develop poor dietary habits, heightening the risk of malnutrition. On the other hand educational attainment is often linked to income,^(45)^ which plays a crucial role in determining access to healthy foods. By enhancing educational opportunities, nutritional outcomes can be improved, as individuals with higher education are typically more knowledgeable about maintaining good self-care and nutrition. However, in this study, the connection between undernutrition and lower economic status was not examined, as no statistical link was found between average monthly income and undernutrition. Nevertheless, education alone does not ensure improved practices, as social and environmental factors also significantly influence behaviour. Therefore, interventions must go beyond education to address these wider determinants.^(46,47)^

Diabetes patients with at least one comorbid condition showed more than two times more likely to be undernourished. The occurrence of comorbid conditions in patients with diabetes significantly increases the risk of undernutrition. Conditions such as hypertension, cardiovascular disease, and kidney dysfunction can impair nutritional intake, disrupt nutrient absorption, and alter metabolic processes, all of which contribute to a higher likelihood of malnutrition in these patients.^(48,49)^ This association might be explained by a diabetes patient with comorbidity having difficulty controlling blood sugar. If blood sugars are high, it can make the patient urinate frequently, and this results in dehydration as a possible cause of weight loss. Muscle breakdown can also occur if blood sugars are too high and cells can’t utilise enough insulin to convert glucose into energy. Due to this, the body starts consuming muscle and body fat, resulting in unhealthy weight loss. Additionally, some comorbidity may also cause loss of appetite, diarrhoea, constipation, and vomiting, which results in an abnormal loss of weight among patients.

Likewise, the relationship between uncontrolled blood sugar levels and undernutrition can be explained by impaired glucose regulation, which affects nutrient absorption and energy balance, resulting in unhealthy weight loss. This, in turn, can worsen nutritional deficiencies, increase health complications, and create a cycle of metabolic and nutritional imbalances that further compromise overall well-being of the patient.

### Strengths of the study

This study is one of the few to investigate nutritional status and comorbid conditions, areas that have been underexplored in previous research conducted in Ethiopia and similar settings.

### Limitations of the study

The cross-sectional design and small sample size of this study limit its generalizability. Moreover, nutrition was assessed solely using BMI, without considering other influencing factors or the impact of anti-diabetic medications. Recall bias may have influenced the responses regarding food intake. The study also did not account for nutritional expertise or genetic predispositions to under- or over-nutrition. Additionally, the lack of hypertension measurement contributed to the low prevalence reported, representing another limitation.

## Conclusion

This study evaluates the prevalence of undernutrition and its predictors among adult outpatients living with diabetes. Our findings indicate a high prevalence of concordant comorbidities among this population. Specifically, undernutrition was significantly predicted by the presence of comorbidities, alcohol consumption, educational level, and uncontrolled blood sugar levels. Preventing comorbidities in adult outpatients living with diabetes is a vital aspect of comprehensive diabetes care, requiring regular screening, early detection, and prompt management. Tailored nutrition counselling promoting sustainable lifestyle changes, such as adopting healthier eating habits, is strongly recommended. Equally important is strengthening healthcare professionals’ ability to identify and manage diabetes-related comorbidities effectively. Furthermore, the study’s results are applicable to similar populations and settings, with due consideration given to sample representativeness and the study’s limitations.

## Data Availability

The data that support the findings of this study are available from the corresponding author upon reasonable request.
